# Purinergic Cooperation Between P2Y_2_ and P2X7 Receptors Promote Cutaneous Leishmaniasis Control: Involvement of Pannexin-1 and Leukotrienes

**DOI:** 10.3389/fimmu.2018.01531

**Published:** 2018-07-09

**Authors:** Maria Luiza Thorstenberg, Marcos Vinícius Rangel Ferreira, Natália Amorim, Claudio Canetti, Fernanda B. Morrone, José Carlos Alves Filho, Robson Coutinho-Silva

**Affiliations:** ^1^Laboratório de Imunofisiologia, Instituto de Biofísica Carlos Chagas Filho, Universidade Federal do Rio de Janeiro, Rio de Janeiro, Brazil; ^2^Laboratório de inflamação, Instituto de Biofísica Carlos Chagas Filho, Rio de Janeiro, Brazil; ^3^Laboratório de Farmacologia Aplicada, Pontifícia Universidade Católica do Rio Grande do Sul, Porto Alegre, Brazil; ^4^Departamento de Farmacologia, Faculdade de Medicina de Ribeirão Preto, Universidade de São Paulo, Ribeirão Preto, Brazil

**Keywords:** *Leishmania amazonensis*, LTB_4_, PANX-1, P2Y_2_, P2X7

## Abstract

The release of damage-associated molecular patterns, including uridine triphosphate (UTP) and adenosine triphosphate (ATP) to the extracellular milieu is a key component of innate immune response to infection. Previously, we showed that macrophage infection by the protozoan parasite *Leishmania amazonensis*—the etiological agent of cutaneous leishmaniasis—can be controlled by ATP- and UTP-mediated activation of P2Y and P2X7 receptors (activated by UTP/ATP and ATP, respectively), which provided comparable immune responses against the parasite. Interestingly, in context of *Leishmania amazonensis* infection, UTP/P2Y triggered apoptosis, reactive oxygen species, and oxide nitric (NO) production, which are characteristic of P2X7 receptor activation. Here, we examined a possible “cross-talk” between P2Y_2_ and P2X7 receptors, and the requirement for pannexin-1 (PANX-1) in the control of *L. amazonensis* infection in mouse peritoneal macrophages and *in vivo*. UTP treatment reduced *L. amazonensis* parasite load, induced extracellular ATP release [which was pannexin-1 (PANX-1) dependent], and triggered leukotriene B_4_ (LTB_4_) production in macrophages. UTP-induced parasite control was blocked by pharmacological antagonism of P2Y_2_ or P2X7 receptors and was absent in macrophages lacking P2X7 or PANX-1. In addition, ATP release induced by UTP was also inhibited by PANX-1 blocker carbenoxolone, and partially reversed by inhibitors of vesicle traffic and actin cytoskeleton dynamics. *In vivo*, UTP treatment reduced footpad and popliteal lymph node parasite load, and the lesion in wild-type (WT) mice; fact not observed in P2X7^−/−^ mice. Our data reveal that P2Y_2_ and P2X7 receptors cooperate to trigger potent innate immune responses against *L. amazonensis* infection.

## Introduction

According to the World Health Organization, leishmaniasis is the second most prevalent cause of parasite-associated deaths, being responsible for an estimated 30,000 deaths annually (1). Three main manifestations of disease can occur, namely cutaneous, mucocutaneous (affecting the skin and mucous membranes), and visceral leishmaniasis (2). Among all the diagnosed cases of leishmaniasis, 75% are of the cutaneous forms, which can evolve into mucocutaneous or disseminated, two more severe form of the disease characterized by parasite migration to secondary sites and the formation of metastatic lesions. Cutaneous leishmaniasis is most frequently displayed as ulcerating skin lesions at the site of the sand fly bite and are caused by *L. amazonensis, L. major, L. braziliensis*, and *L. guaynensis* species (2).

*Leishmania* parasites are transmitted by an infected sandfly vector as metacyclic promastigote forms, which establish the infection in phagocytic cells (i.e., macrophages, neutrophils, and dendritic cells), where they proliferate as obligate intracellular amastigotes within phagolysosome compartments (3). Early during *Leishmania* infection, phagocytes recruited to the infection site recognize pathogen-associated molecular patterns, which leads to the release of damage-associated molecular patterns (DAMPs)—including nucleotides—such as adenosine triphosphate (ATP), uridine triphosphate (UTP), and their metabolites ADP and UDP—that are involved in host resistance (4, 5). DAMPs release orchestrate the inflammation, and which include both cell-dependent mechanisms (i.e., phagocytosis and cytotoxicity) and the secretion of inflammatory mediators to the extracellular milieu (6); ATP and UTP found in the extracellular environment activate immune cells, allowing effective microbicidal responses against intracellular pathogens (7).

Nucleotides reach the extracellular space through vesicular secretion and pannexin-1 (PANX-1) membrane channels, and the PANX-1 mediated release of ATP and UTP from apoptotic cells is required for phagocytic cells recruitment during inflammation and apoptotic cell clearance (8). Extracellular UTP and ATP (eUTP and eATP, respectively) exert their effects on phagocytic cells *via* surface P2 receptors that are classified into eight metabotropic P2Y receptors (P2Y_1,2,4,6,11–14_) and seven ionotropic P2X (P2X1–7) receptors (9, 10). In macrophages, the P2X7 receptor stimulates the production of reactive oxygen species (ROS) and NO, and it is involved in the activation of caspases and phospholipases (11), as well as on apoptosis (12) and the processing and secretion of the pro-inflammatory interleukins—IL-1β and IL-18 (13).

Given that extracellular nucleotide release is likely to occur in a wide range of conditions, P2X and P2Y receptors are involved in a range of immunological reactions and have been widely studied in pathological settings, including sepsis (14), *Toxoplasma gondii* infection (15), and leishmaniasis (16). Importantly, immune cells can have variable sensitivity to extracellular nucleotides, since treatment with the pro-inflammatory cytokines interferon-γ (IFN-γ) and tumoral-necrosis factor-α (TNF-α) renders phagocytes more sensitive to eATP than in resting cells (17, 18). The reason for this increased immune response to eATP is unclear. However, it may be due to higher levels of expression or activity of P2 receptors (19, 20), which is correlated with eATP triggered increases in the levels of ROS, NO, IL-1β, and CC chemokine ligand-2 in intracellular infections in comparison with uninfected cells (21–23).

Our group has reported the involvement of extracellular nucleotides such as eATP and eUTP in the control of different parasitic diseases. In previous studies, we showed that ATP secreted after cell lysis can control the survival of the protozoan parasites *Plasmodium chabaudi* and *Toxoplasma gondii* (24, 25), in a mechanism involving ROS production (26). We also showed that *Leishmania amazonensis* infection positively modulates the expression of P2X7, P2Y_2_, and P2Y_4_ receptors in macrophages (19, 20), and that the activation of P2 receptors by nucleotide secretion is likely to represent a physiological mechanism for parasitism control (5), since UTP and ATP have crucial roles in restraining the proliferation of *L. amazonensis* (19, 20, 27). P2X7 receptor activation induces resistance to *L. amazonensis* infection in macrophages, *via* a mechanism involving LTB_4_ production (5). Interestingly, P2Y activation by eUTP in *L. amazonensis* infected macrophages triggered effects traditionally associated with ATP-mediated P2X7 activation, such as the apoptosis, NO, and ROS production, in a calcium-dependent manner (19). These data suggest the existence of a “cross-talk” between ATP- and UTP-mediated responses during *Leishmania* infection.

In this study, we examined the possible mechanisms involved in the control of parasite load by P2Y_2_ receptor agonists (UTP/ATP). Assuming that different P2 receptors are upregulated in macrophages infected with *L. amazonensis*, we evaluated the “cross-talk” between P2 receptors of high and low affinity to ATP, as well P2Y_2_, and P2X7 receptors, both *in vitro* and *in vivo*, using knockout mouse models and pharmacological tolls. Our data reveal the existence of a system whereby eUTP/ATP stimulation amplifies ATP secretion by infected macrophages—*via* the activation of PANX-1 channels—culminating in further P2X7 receptor ligation, generating LTB_4_, which boosts the immune response against *L. amazonensis*.

## Materials and Methods

### Reagents

Uridine triphosphate, ATP, UTP-λ-S, Dulbecco’s modified Eagle’s medium (DMEM), and 199 medium were purchase from Sigma-Aldrich (St. Louis, MO, USA). A740003, ARC118925, and U73122 were from Tocris (UK). The ATP determination Kit was from Life probes (Waltham, MA, USA). LTB_4_ levels were determined using the EIA Kit from Cayman (Ann Arbor, MI, USA).

### Mice

The following mouse strains were used in this study: wild type BALB/c and C57BL/6, as well as P2X7 receptor knockout (P2X7^−/−^; in the C57BL/6 background), as well as Pannexin-1 deficient (PANX-1^−/−^; in the C57BL/6 background), and 5-lipoxygenase (5-LO)-deficient mice (5-LO^−/−^; in the background SV129) mutant strains, originally from the Jackson Laboratory (Sacramento, CA, USA). Mice were maintained at the Animal House for Transgenic Mice of the Federal University of Rio de Janeiro (UFRJ, RJ, Brazil), at 22°C, and in a 12-h light/dark cycle. Mice aged between 8 and 12 weeks were used in all experiments. The procedures for the care and use of animals were according to the guidelines of the Brazilian College of Animal Experimentation (COBEA). The animal experimentation protocols used in this study were approved by the Ethics Committee on the Use of Animals (CEUA) from the Institute of Biophysics Carlos Chagas Filho (IBCCF, UFRJ; document no. 077/15).

### Parasite Culture

*Leishmania amazonensis* (MHOM/BR/Josefa) parasites were maintained by serial passages from BALB/c mouse lesions, to preserve virulence. Amastigotes isolated from mouse lesions were allowed to transform into axenic promastigotes forms by growth at 24°C, for 7 days, in 199 medium supplemented with 10% heat inactivated fetal bovine serum (FBS; Gibco BRL), 2% male human urine, 1% l-glutamine, and 0.25% hemin. For both *in vitro* and *in vivo* infection experiments, promastigotes were used in the late stationary phase of growth.

### Peritoneal Macrophage Culture and Infection

Mouse peritoneal macrophages were obtained by peritoneal washes with cold phosphate buffer saline (PBS). Cells were allowed to adhere for 1 h (in DMEM supplemented medium, at 37°C, with 5% CO_2_) and then washed gently with PBS (twice), to remove the non-adherent cells. Then adherent cells were then cultured in DMEM supplemented (10% FBS and 100 U penicillin/streptomycin), at 37°C (and 5% CO_2_). After 24 h, macrophages were allowed to interact for 4 h with *L. amazonensis* promastigotes, and then non-adhered parasites were removed by extensive washing with PBS. Infected cultures were maintained in supplemented DMEM medium until further use.

### Nucleotide and Inhibitor Treatments

The treatment of infected cells with nucleotides or inhibitors was performed at least 48 h post-infection. Infected macrophages were treated with 25 nM A740003, 2 µM U73122, or 10 µM ARC118925 for 30 min, and then stimulated with 100 µM UTP, 100 µM UTP-λ-S, 50, 100, or 500 µM ATP for a further 30 min, at 37°C (and 5% CO_2_). After treatment, cell monolayers were washed with PBS and DMEM supplemented.

### *In Vitro* Parasite Load Analysis

The intracellular parasite load was analyzed 24 h after nucleotide treatment, by light microscopy. Cells were plated onto 24-well glass coverslips and treated as described above. After 24 h of incubation with nucleotides, cell supernatants were discarded and samples were fixed with 4% paraformaldehyde for 10 min and then stained with panoptic stain (Laborclin^®^, PR, Brazil), according to the manufacturer’s instructions. Slides were examined in a Primo Star light microscope (Zeiss, Germany), using a 100× oil-immersion objective. Images were acquired using a Bx51 camera (Olympus, Tokyo Japan) operated by the Cell^F software and were used to estimate the “infection index,” which represents the overall infection load. The “infection index” was calculated using following formula:

$$\%\text{ Infection = }\frac{\text{(total number of infected cells }\times \text{ 100)}}{\text{Total number of cells}}$$

Infection index=(% of infection×total number of amastigote)÷100Total number of cells

### Mouse Infection

Female WT and P2X7^−/−^ mice (8–12 weeks old) were infected subcutaneously in the footpad with 10^6^ *L. amazonensis* promastigotes resuspended in PBS. After 7 days post-infection (d.p.i.), infected footpads were inoculated twice a week, for 3 weeks (total of six administrations) with 20 µL of 1 mM UTP (pH = 7.2) or PBS. The “swelling” (=thickness of the infected footpad − thickness of the uninfected footpad from the same mouse) was measured weekly using a traditional caliper (Mitutoyo^®^). The swelling (thickness) was evaluated before the final UTP injection, during the course of treatment. Forty-eight hours after the last injection (26 d.p.i.), the animals were subjected to euthanasia, and selected organs (footpad and popliteal lymph nodes) were removed for further analysis, as described below.

### Parasite Load in Mouse Tissues

To estimate the parasite load in infected tissues, the number of living *L. amazonensis* parasites was determined in the infected footpad and popliteal lymph nodes by the limiting dilution assay (LDA), as described previously. After animal euthanasia, footpads and lymph nodes were collected, weighed, the cells from the footpad and lymph node were dissociated using a cell strainer (BD^®^), in M199 medium. Tissue debris was removed by centrifugation at 150 *g*, and then cells were separated by centrifugation at 2,000 *g* for 10 min and resuspended in complete M199.

Aliquots of 50 µl from each cell suspension (at an initial density of 0.25^−1^ × 10^6^ cells/ml) were plated into 96-well, flat-bottom microtiter plates (BD^®^, USA), in serial 4:1 dilutions in complete M199 medium, and in triplicates. Samples were cultured at 26–28°C and, after a minimum of 7 days, wells were examined by phase-contrast microscopy in an inverted microscope (NIKON TMS, JP), and scored as “positive” or “negative” for the presence of parasites. Wells were scored as “positive” when at least one parasite was observed per well, and the parasite load (*L. amazonensis* count per footpad) was estimated according to the highest dilution in which parasites could still be detected, as described earlier (28).

### Cellularity Analysis

To analyze the cellular frequency in the footpad, tissue was placed in 15 ml falcon BD^®^ tubes and digested for 1 h with 1 mg/ml collagenase (Sigma^®^) in RPMI medium containing 15% FBS and 100 U penicillin/streptomycin. Tubes were vortexed at 15 min intervals to accelerate digestion. Then, footpad cells were dissociated in a cell strainer and resuspended in 1 ml of RPMI medium and centrifuged at 120 *g* for 10 min, to remove cell debris. To obtain single cell suspensions from lymph nodes, they were removed and dissociated using a 40-µm cell strainer, centrifuged at 120 *g* for 10 min, and resuspended in complete RPMI. The cell viability (exclusion of 0.2% Trypan Blue) and leukocyte frequency were measured by phase-contrast microscopy, in an inverted microscope (NIKON TMS, JP).

### ATP Release Assay

Adenosine triphosphate release was measured using a luciferase-based assay. Peritoneal macrophages and popliteal lymph node cells (in single-cell suspensions prepared as described above) were cultured overnight at a density 10^6^ cells/well, in 96-well plates. Infected and uninfected macrophages were subjected to a 30-min “pulse” with UTP (100 µM) in serum-free DMEM medium, and then cells were removed by centrifugation at 2,000 *g* for 10 min. The measurements of eATP from the culture supernatant were performed using the ATP determination kit (Life^®^ Probes) by real-time luminometry, according to the manufacturer’s instructions. The luminescence of samples plated onto black 96-well plates was read in a SpectraMax^®^M5/M5e Multimode Plate Reader (Molecular Devices), and results were expressed as pmol of eATP/10^6^ cells, at different time-points.

### Leukotriene B_4_ (LTB_4_) Release Assay

To measure the LTB_4_ released in cell supernatants, macrophages from WT and P2X7^−/−^ mice were plated in 96-well plates and infected as described above. After 48 h of infection, the supernatant was removed, and new medium without FBS was added. Then, cultures were treated with 100 µM eUTP for 30 min, washed, and incubated for 24 h at 37°C (and 5% CO_2_). After incubation, cultures were centrifuged at 120 *g* for 5 min, and supernatants were collected for LTB_4_ detection using an enzyme immunoassay-Cayman Chemical (An Arbor, MI, USA). The results were measured in an enzyme-linked immunosorbent assay (ELISA) plate reader at a wavelength between 405 and 420 nm. The supernatants were stored at −80°C until further analysis.

### Cytokine Assays

Single-cell suspensions from the popliteal lymph node removed from P2X7^−/−^or WT mice 26 days post-infection were prepared aseptically (as described above—see “Cellularity analysis”), diluted in complete RPMI-1640 medium (Gibco^®^) for a final concentration of 10^6^ cells/well (96-well plates; 200 µl/well) previously stimulated with plate-bound α-CD3 mAb (2 µg/ml; BD^®^ Biosciences). After 72 h of culture at 37°C (in 5% CO_2_), cells were removed by centrifugation at 2,000 *g* for 8 min, and the levels of IL-1β and IL-17 in culture supernatants were determined by ELISA, using commercial kits (R&D Systems, Catalog numbers DY 401, Minneapolis, MN, USA).

### Statistical Analysis

Statistical analyses were performed by Student’s *t*-test, and one-way analysis of variance (ANOVA) coupled with Tukey’s multiple comparison *post hoc* test, using the Prism 5.0 software (GraphPad Software, La Jolla, CA, USA). Values of *P* < 0.05 were considered statistically significant.

## Results

### Uracil and Adenine Nucleotides Are Involved in *L. amazonensis* Infection Control in Macrophages

Adenosine triphosphate is considered an important and the most ancient DAMP in the immune system, mediating physiological responses *via* the activation of cell surface P2X and P2Y receptors (29). Here, we evaluated a possible “cross-talk” between P2Y_2_ and P2X7 mediated by eATP, based on the assumption that differential levels of eATP have distinct effects on P2 receptors depending on their concentration at the active site. As a rule, low eATP levels (in the micromolar range EC_50_ ≤ 25 µM) are required to activate the P2Y_2_ receptor (10), while moderate to high eATP levels (EC_50_ ≥ 100 µM) are necessary to activate the P2X7 receptor (30). However, in context of inflammation, the eATP levels required for activation of P2X7 receptor-mediated immune responses can be lower, but the mechanism behind this increased sensitivity to eATP is poorly understood.

**Figure 1:**
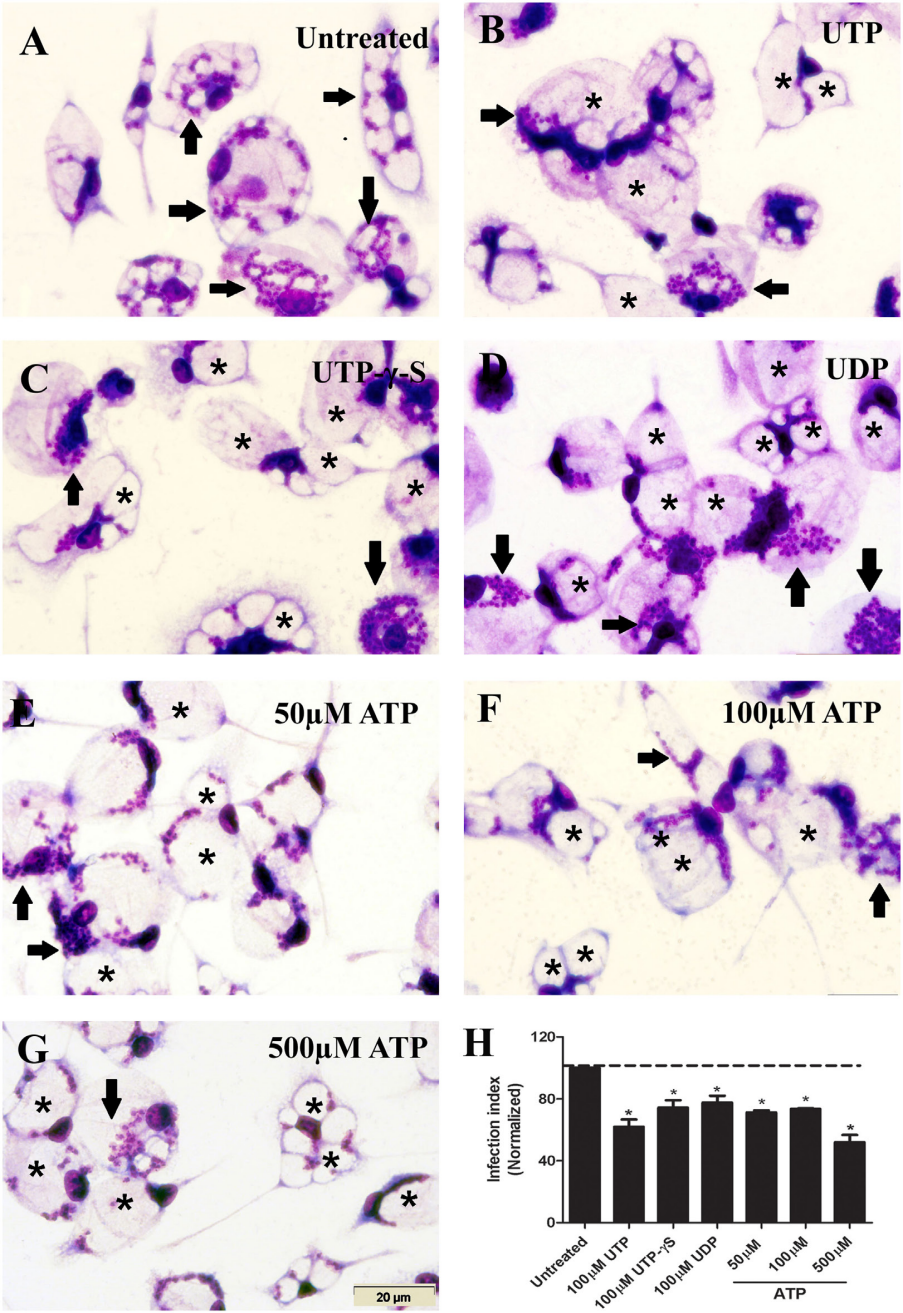
Extracellular nucleotides reduce the parasite load in macrophages infected with *Leishmania amazonensis*. **(A–H)** Infected peritoneal macrophages were kept untreated **(A)**, or were treated for 30 min with 100 µM UTP **(B)**, 100 µM UTP-γ-S **(C)**, 100 µM UDP **(D)**, 50 µM adenosine triphosphate (ATP) **(E)**, 100 µM ATP **(F)**, and 500 µM ATP **(G)**. After 30 h, cells were fixed, stained with Panoptic, and observed by light microscopy. Arrows indicate vacuoles with *L. amazonensis* amastigotes, and asterisks indicate empty vacuoles. Scale bar, 20 µm. **(F)** The effect of nucleotides on infection was quantified by determining the “infection index” (% of infection × number of amastigote/total number of cells/100; normalized to the untreated), by direct counting under the light microscope. Data represent mean ± SEM of three independent experiments performed in triplicate, with pools of cells from 4 to 5 animals. *P < 0.05 relative to the untreated group (by one-way analysis of variance followed by Tukey’s test).

Initially, we confirmed the anti-parasitic effects induced by uridine nucleotides (UTP or UDP) in macrophages infected with *L. amazonensis* (19). Treatment with 50–500 µM ATP (Figures 1E–G) and UTP-γ-S (not hydrolyzable UTP) (Figure 1C) reduced the infection index (an estimate of parasite load) in macrophages infected with *L. amazonensis*. These data suggest the participation of P2Y_2_ in initiate the infection control (at 50–100 µM ATP); however, infection also reduced at 500 µM ATP concentration, which involves the activation of P2X7 receptor.

### P2Y_2_ Receptor Activation Is Required for Both eUTP- and eATP-Mediated Anti-Leishmanial Effects *In Vitro*

**Figure 2:**
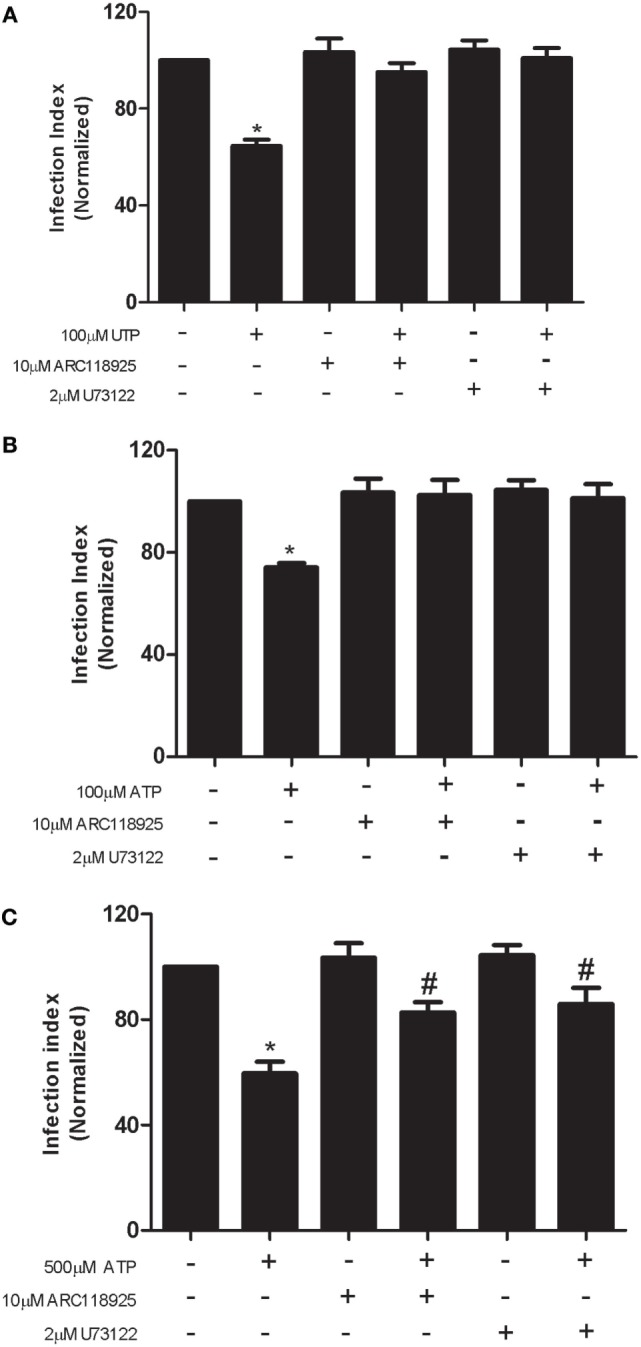
The UTP- and adenosine triphosphate (ATP)-stimulated control of *Leishmania amazonensis* infection in macrophages depends on P2Y_2_ receptor activity. **(A–C)** Infected macrophages were untreated or treated with 10 µM ARC118925 and 2 µM U73122 for 30 min (to inhibit P2Y_2_ receptors and phospholipase C activity, respectively), and then stimulated for 30 min with UTP [100 µM; **(A)**] or ATP [100 µM, **(B)** or 500 µM, in **(C)**]. Cells were fixed and stained 30 h after the end of the stimulation period. The parasite load in infected macrophages was quantified as the “infection index” (% of infection × number of amastigote/total number of cells/100; normalized to the untreated), determined by direct counting from light microscopy images. Data represent mean ± SEM values of three independent experiments, performed in triplicates, with pooled cells from 4 to 5 animals. *P < 0.05 (relative to the untreated group) or ^#^*P* < 0.05 (relative to the treated group) (by one-way analysis of variance followed by Tukey’s test).

Our previous studies indicate that P2Y_2_ expression and function are significantly upregulated in macrophages infected with *L. amazonensis* (19). The P2Y_2_ receptor, which responds equipotently to ATP and UTP, is upregulated in inflamed or damaged tissues (31, 32). Upon activation, P2Y_2_ stimulates canonical Gqα signaling, increasing phospholipase C activity and leading to inositol 1,4,5-trisphosphate and diacylglycerol production, which lead to in intracellular Ca^2+^ release and protein kinase C activation, respectively (9, 10). The participation of P2Y_2_ receptors in eUTP- and eATP-mediated anti-leishmanial effects was addressed by pre-treatment of infected cells with the selective P2Y_2_ antagonist ARC118925 or with the phospholipase C inhibitor U73122 (which blocks signaling downstream of the P2Y_2_), before treatment with nucleotides. We observed that pretreatment with either ARC118925 or U73122 blocked fully (100 µM UTP/ATP) (Figures 2A,B) or partially (500 µM ATP) (Figure 2C) the reduction in parasite load triggered by UTP and ATP, in macrophages infected with *L. amazonensis*. These results indicate that P2Y_2_ receptor signaling is required for both ATP- and UTP-mediated *L. amazonensis* infection control in macrophages, *via* intracellular calcium mobilization dependent on phospholipase C.

### The Anti-*Leishmania* Effects Attributed to P2Y_2_ Receptor Activation Depend on P2X7 Receptors

P2Y_2_ receptors can be activated by eATP as well as eUTP, and *L. amazonensis* infection increases the levels of P2Y_2_ in macrophages (19). Thus, the dependency of both eUTP- and eATP-mediated leishmania infection control on P2Y_2_ receptor activity (Figure 2) could be due to direct activation of this receptor by either nucleotide. Alternatively, eUTP could reduce the parasite load in macrophages indirectly, by increasing extracellular eATP levels, which would then trigger P2X7 activation.

**Figure 3:**
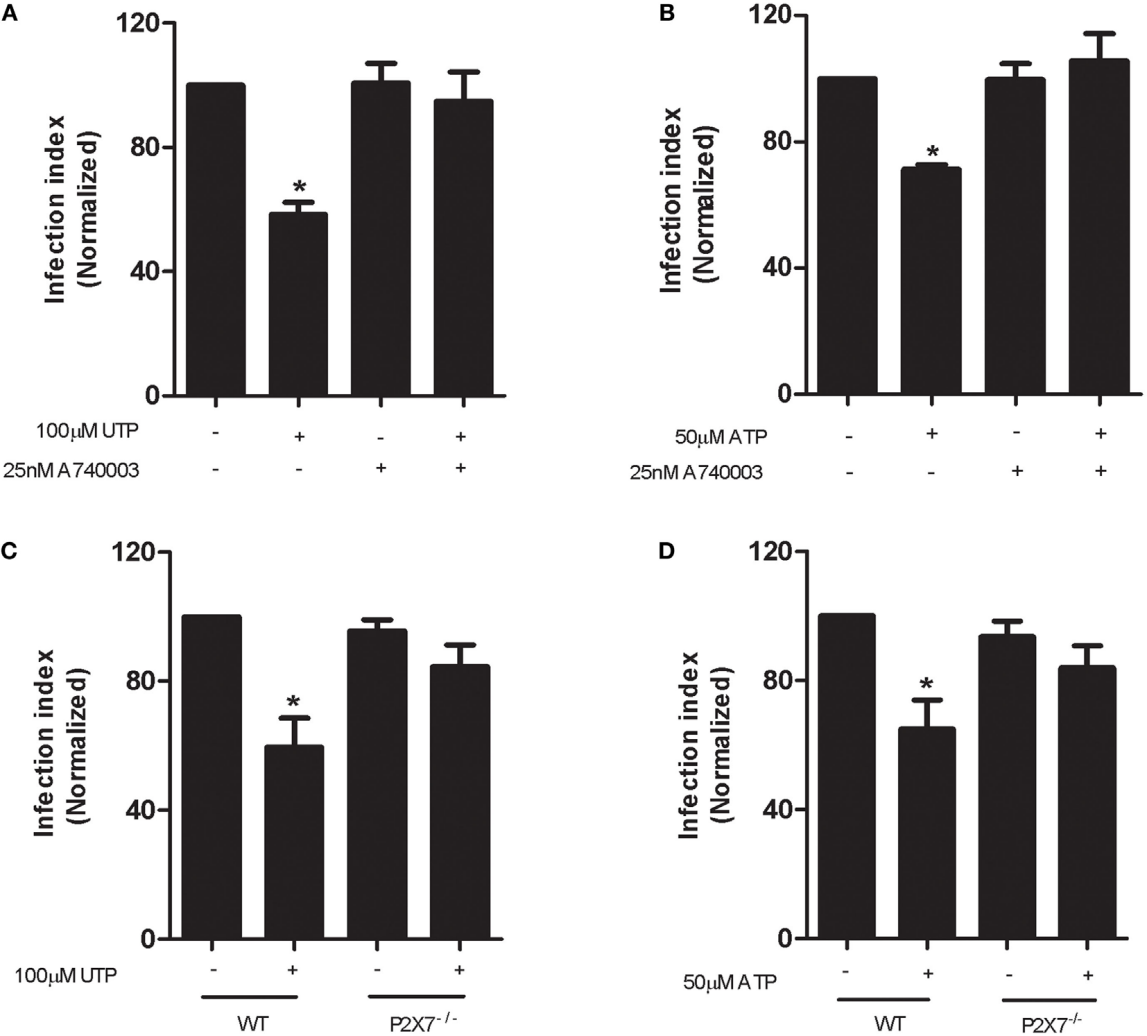
P2Y_2_ and P2X7 receptors cooperate in the uridine triphosphate (UTP)-stimulated control of *Leishmania amazonensis* infection in macrophages. Peritoneal macrophages from WT **(A,B)** and P2X7^−/−^ **(C,D)** mice were infected with stationary-phase *L. amazonensis* promastigotes and then treated, 48 h post-infection, with the P2X7 receptor antagonist A740003 (25 nM) for 30 min **(A,B)**, followed by stimulation with 100 µM UTP **(A–C)** or 50 µM adenosine triphosphate **(B–D)** for 30 min. Infected macrophages were fixed 30 h posttreatment, stained with Panoptic, and the parasite load in infected macrophages was quantified as the “infection index” (% of infection × number of amastigote/total number of cells/100; normalized to the untreated), by direct counting from light microscopy images. Data represent mean ± SEM values of *n* = 3 experiments performed in triplicates, with pooled cells from 4 to 5 animals. *P < 0.05 relative to the untreated group (by one-way analysis of variance followed by Tukey’s test).

To determine if P2X7 receptor activation was required for the eUTP-mediated control of *L. amazonensis* infection, we treated cells with the selective P2X7 receptor antagonist A740003, before stimulation with eUTP. Interestingly, we observed that P2X7 receptor inhibition completely blocked both UTP- and ATP-mediated anti-parasitic effects (Figures 3A,B), similarly to P2Y_2_ receptor antagonism and PLC inhibition. In addition, we evaluated the anti-leishmanial effects of eUTP and eATP in infected macrophages from mice lacking the P2X7 receptor (P2X7^−/−^ mice). Neither UTP nor ATP were capable of reducing the infection index in P2X7^−/−^ macrophages infected with *L. amazonensis* (Figures 3C,D). Taken together, the pharmacological inhibition and genetic knockout data indicate that the contribution of the P2Y_2_ receptor to leishmania infection control is ultimately dependent on a functional P2X7 receptor.

### eUTP Induces ATP Secretion Through Pannexin-1 Channels in Infected Macrophages

**Figure 4:**
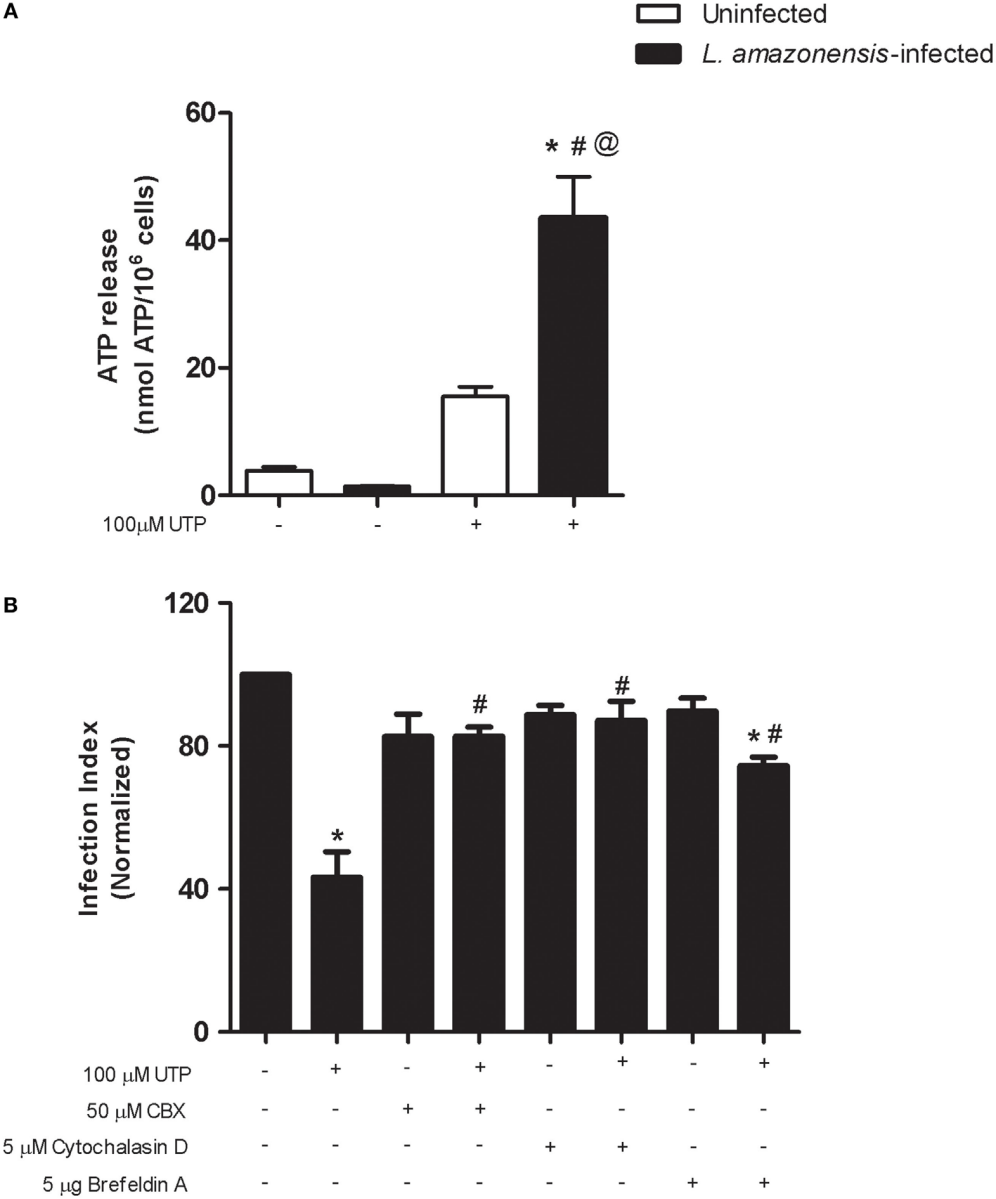
Uridine triphosphate (UTP) treatment induces adenosine triphosphate (ATP) release by macrophages infected with *Leishmania amazonensis*. Peritoneal macrophages WT mice were infected with stationary-phase *L. amazonensis* promastigotes and then stimulated, 48 h post-infection, with UTP in serum-free medium, for 30 min. **(A)** Following stimulation with 100 µM UTP, the ATP levels in the supernatants of uninfected (white bars) and infected (black bars) cells were measured using a luciferase-based assay. **(B)** Before stimulation with UTP, infected cells were treated (for 30 min) with one of the following inhibitors: carbenoxolone (Pannexin-1 inhibitor, at 50 µM), cytochalasin D (exocytosis inhibitor, at 5 µM), and Brefeldin A (hemichannels and vesicular traffic inhibitor, at 5 µg/ml). Then, samples were fixed and stained with Panoptic, 30 h post-stimulation, and the “infection index” (% of infection × number of amastigote/total number of cells/100; normalized to the untreated) was determined by direct counting from light microscopy images. Data represent mean ± SEM values of *n* = 3 experiments performed in triplicates, with pooled cells from 4 to 5 animals. *P < 0.05 **(A)** uninfected vs. infected group, ^#^*P* < 0.05 infected vs. infected treated group, and ^@^*P* < 0.05 vs. uninfected treated vs. infected treated group. **(B)** *P < 0.05 infected vs. infected treated group, and ^#^*P* < 0.05 infected treated (Brefeldin A and UTP) vs. infected UTP treated group (by one-way analysis of variance followed by Tukey’s test).

In immune cells, including macrophages, PANX-1 molecules form gap junction-like structures that function primarily in transferring intracellular molecules such as ATP to the extracellular space (33). In some cell types, the activation of P2Y_2_ receptors results in ATP release *via* PANX-1 pores, and this mechanism is likely to operate in diverse cell types (8). To evaluate if this mechanism operates during *L. amazonensis* infection, we examined the levels of extracellular ATP in the supernatant of uninfected and infected macrophages after UTP treatment. The treatment of uninfected macrophages with UTP increased the extracellular ATP levels (from 3.8 ± 0.6 to 15.5 ± 0.1 nmol ATP/1.0 × 10^6^ cells), when compared with the untreated control (Figure 4A). This effect was more pronounced in infected macrophage cultures, where UTP treatment increased the extracellular ATP levels to 44 ± 7 nmol ATP/10^6^ cells, compared with 1.4 ± 0.1 nmol ATP/10^6^ cells, in untreated cells (Figure 4A).

We showed previously that UTP treatment triggers apoptosis in macrophages infected with *L. amazonensis* (19). In early apoptotic cells, ATP is released to the extracellular milieu via vesicles and PANX-1 channels (8) Thus, we used pharmacological inhibition to examine two potential mechanisms for P2Y_2_-regulated ATP release by *L. amazonensis* infected macrophages, namely the vesicle-mediated ATP exocytosis and the passage of ATP to the extracellular milieu *via* plasma membrane channels. Treatment with the vesicular traffic and hemi-channel blocker brefeldin A partially reversed the reduction in parasite load induced by eUTP (Figure 4B). Importantly, treatment with the PANX-1 inhibitor carbenoxolone (CBX) or with the actin polymerization blocker cytochalasin D fully prevented the reduction in parasite load induced by eUTP, in macrophages infected with *L. amazonensis* (Figure 4B).

**Figure 5:**
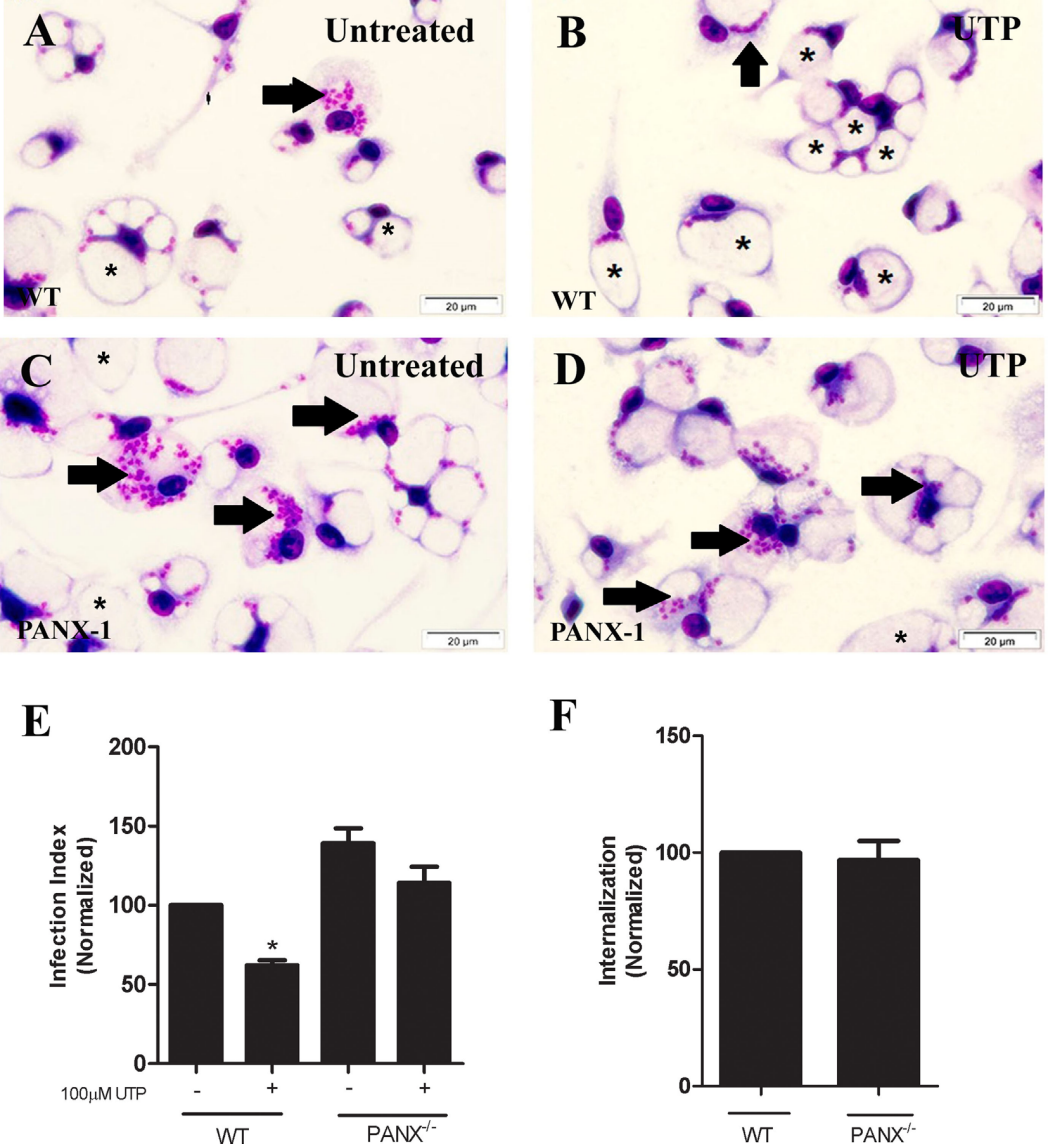
Pannexin channels are essential for the immune response against *Leishmania amazonensis* in macrophages. Infected macrophages from WT and PANX^−/−^ mice were treated with 100 µM uridine triphosphate **(B,D,E)** for 30 min. After 30 h, cells were fixed and stained with Panoptic, for light microscopy imaging **(A–D)**. Arrows indicate vacuoles with *L. amazonensis* and asterisks indicate empty vacuoles. **(E)** The parasite load in infected cells was quantified as the “infection index” (% of infection × number of amastigote/total number of cells/100; normalized to the untreated), by direct counting under the light microscope. **(F)** Parasite load at an earlier time-point (4 h) of infection with *L. amazonensis*, in untreated cells. Data correspond to the mean ± SEM values of *n* = 3 experiments performed in triplicates, with pooled cells from 4 to 5 animals. *P < 0.05 relative to the untreated group (by analysis of variance followed by Tukey’s test).

These results suggest that the ATP release triggered by eUTP in macrophages infected with *L. amazonensis* depends primarily on PANX-1 activity, and on an intact actin cytoskeleton. Therefore, we evaluated further the role of PANX-1 in mediating the immune response induced by UTP in *L. amazonensis* infection, by infecting macrophages from mice lacking pannexin-1 (PANX-1^−/−^). Although macrophages from PANX-1^−/−^ and WT mice were capable of internalizing *Leishmania* promastigotes (Figure 5F), 48 h postinfection the parasite load in cells from PANX-1^−/−^ mice was considerably higher than that in macrophages from WT mice (Figures 5A,C,E). In addition, eUTP-treatment reduced the parasite load in macrophages from WT mice, but not in macrophages from PANX-1^−/−^ animals (Figures 5B–E). Thus, our combined data indicate that P2X7 receptor activation, PANX-1 channels, and ATP release are involved in the control of *L. amazonensis* infection induced by UTP treatment, *in vitro* (Figure 5E).

### LTB_4_ Production Mediated by eUTP Depends on P2X7 Receptor Activity in Infected Macrophages

**Figure 6:**
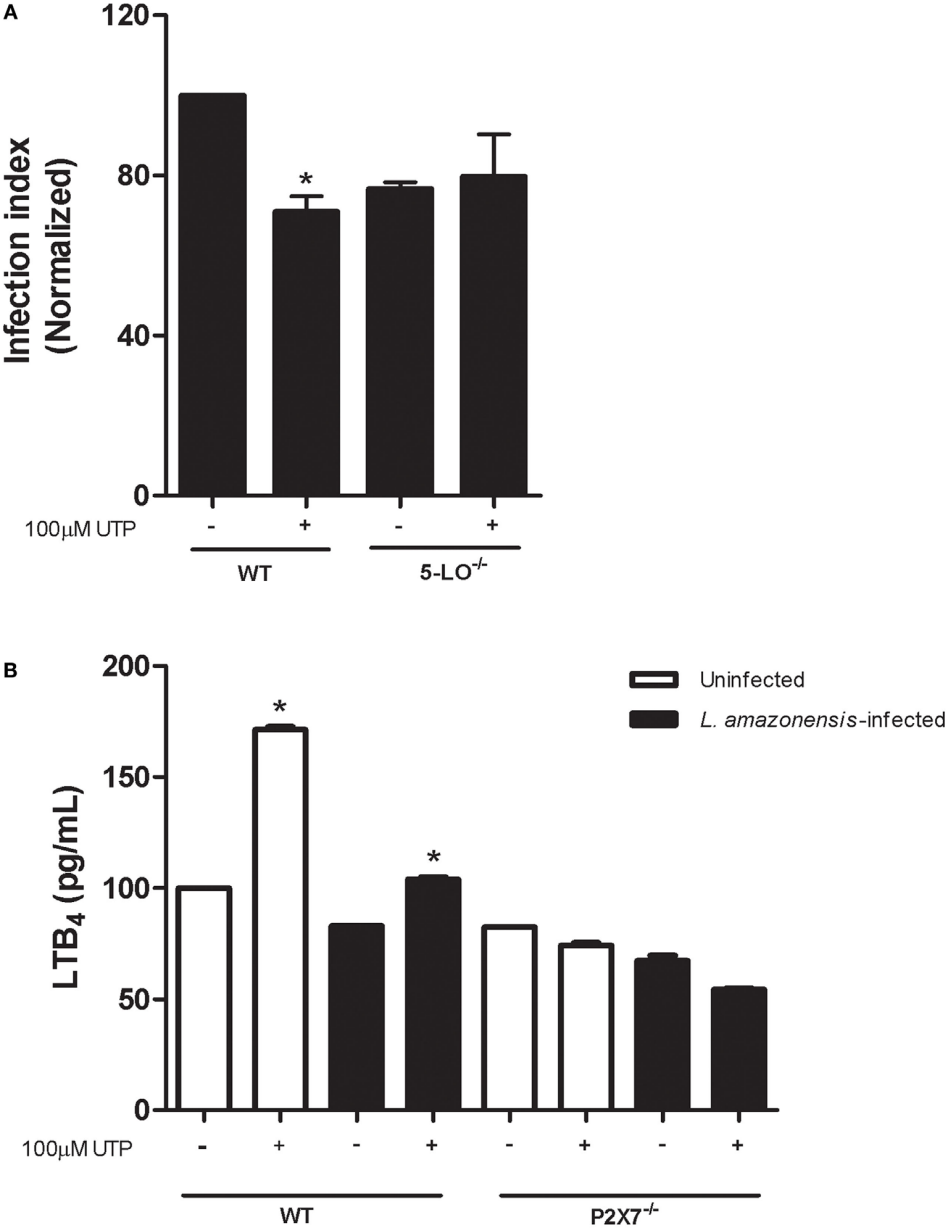
Uridine triphosphate (UTP) treatment induces P2X7-dependent LTB_4_ production in macrophages infected with *L. amazonensis*. **(A)** Infected macrophages form WT and 5-LO^−/−^ mice were treated with 100 µM UTP for 30 min. After 30 h, cells were fixed and stained with Panoptic, for light microscopy imaging. The parasite load in infected cells was quantified as the “infection index” (% of infection × number of amastigote/total number of cells/100, normalized to the untreated), by direct counting under the light microscope. **(B)** Infected or uninfected WT and P2X7^−/−^ macrophages were treated with 100 µM UTP for 30 min. After 24 h of treatment, culture supernatants were harvested and LTB_4_ levels were evaluated by enzyme immunoassay. Data correspond to the mean ± SEM values of *n* = 3 experiments performed in triplicates, or mean ± SEM values of *n* = 3 experiment (normalized to the uninfected WT group), with pooled cells from 4 to 5 animals. *P<0.05 relative to the corresponding untreated group (by one-way analysis of variance followed by Tukey’s test).

LTB_4_ are lipidic mediators synthetized from arachdonic acid, produced by leukocytes during inflammation and triggered leishmanicidal activity on macrophages infected with *L. amazonensis* (5). In previous studies, our group demonstrated that the P2X7 receptor is required for *L. amazonensis* infection control mediated by the LTB_4_ production, in macrophages. Based on these findings and on data indicating that LTB_4_ reduces the parasite load in infected macrophages, we examined the importance of 5-LO—the enzyme that converts arachidonic acid into LTB_4_—in reducing the parasite load mediated by eUTP. We found that *L. amazonensis* infection was not reduced by eUTP treatment in macrophages from 5-LO^−/−^ mice (Figure 6A). In addition, eUTP triggered LTB_4_ production in macrophages infected with *L. amazonensis*, and infected macrophages from mice lacking the P2X7 receptor did not induce LTB_4_ secretion upon eUTP treatment (Figure 6B). These results indicate that eUTP treatment activates 5-LO, triggering the production of LTB_4_ in infected macrophages, *via* a mechanism dependent on the P2X7 receptor.

### UTP Treatment Protects Mice From Leishmaniasis, but Only in the Presence of P2X7

**Figure 7:**
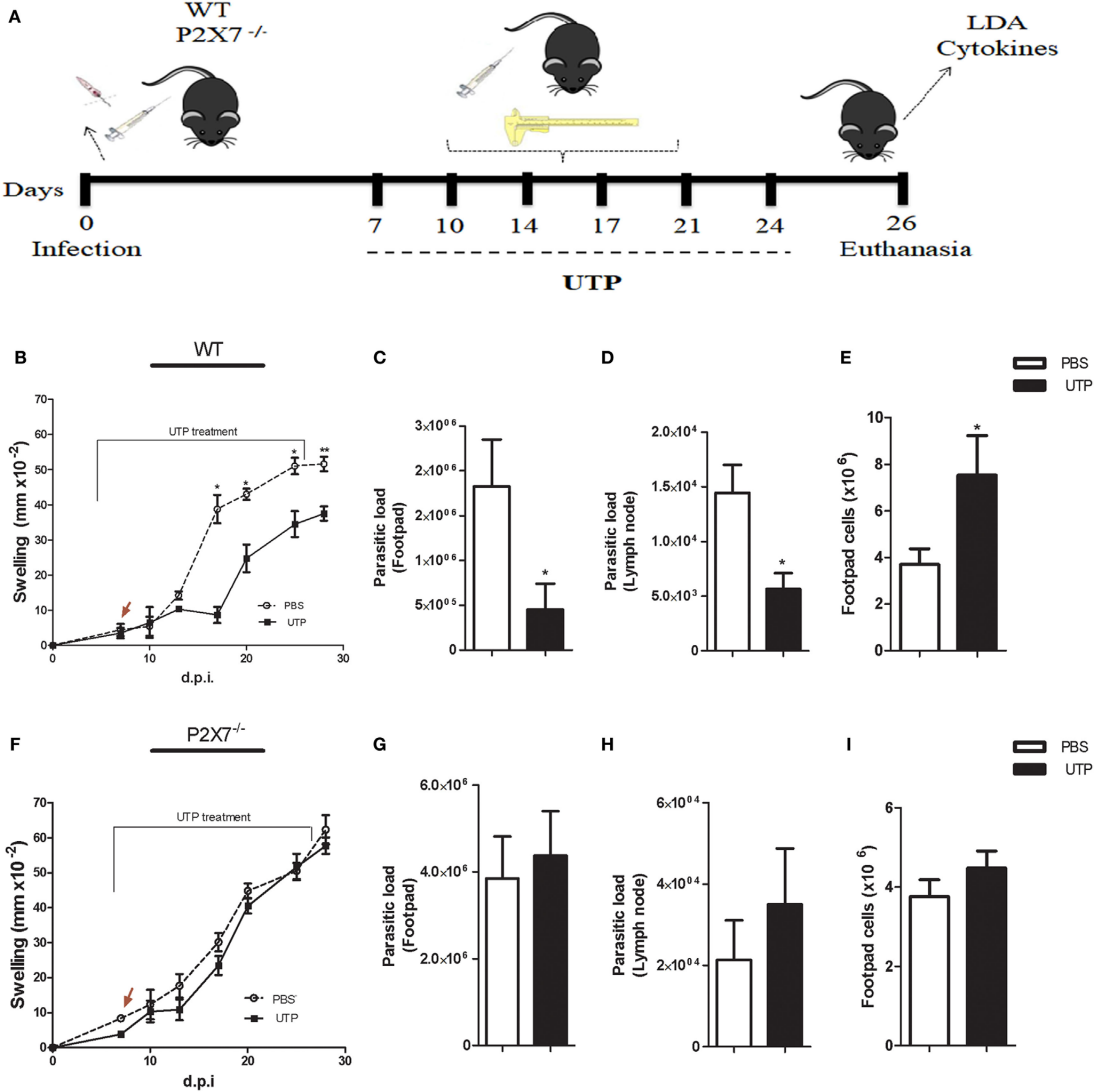

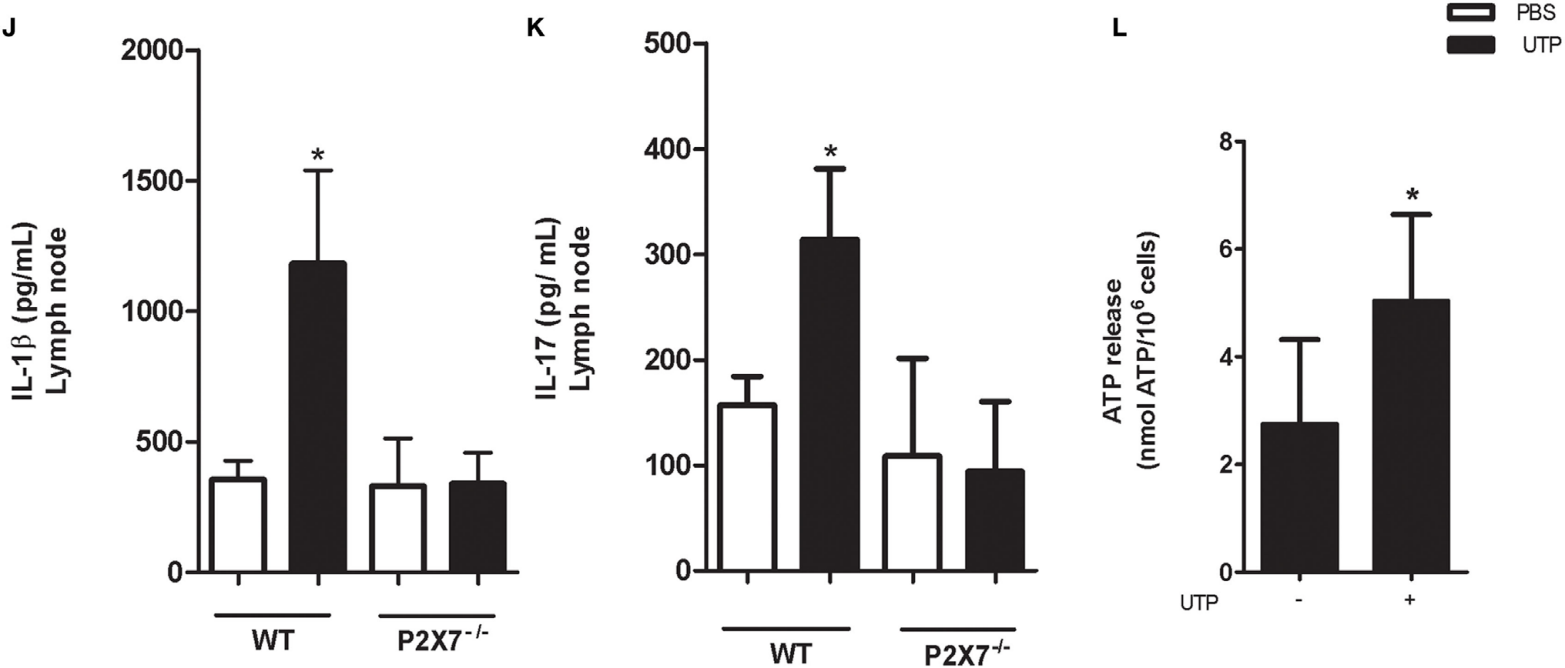
The P2X7 receptor is involved in *Leishmania amazonensis* resistance by P2Y-uridine triphosphate (UTP) activation *in vivo*. **(A)** Schematics showing the design of *in vivo* experiments. WT and P2X7^−/−^ mice (*n* = 6–5 mice/group) were infected subcutaneously in the footpad with 10^6^ *L. amazonensis* promastigotes. From 7 days post-infection (d.p.i.), mice were treated with 1 mM UTP (six doses, injected into the infected footpad twice a week), for 3 weeks (7–24 d.p.i.). The lesion size was determined using a caliper (*n* = 6–5 animals). **(B,F)** Animals were euthanized 26 d.p.i., and the footpad and poplietal lymph node were removed and used for further analysis. **(B–L)** The parasite load in the infected footpad **(C,G)** and lymph node **(D,H)** was determined by a limiting dilution assay (LDA). **(E,I)** The number of infiltrating footpad cells in WT **(E)** and P2X7^−/−^ mice **(I)**. **(J,K)** Levels of the cytokines IL-1β **(J)** and IL-17 **(K)** (determined by ELISA) produced by lymph node cells in single-cell suspensions, after 72 h of stimulation with an α-CD3 antibody. **(L)** Adenosine triphosphate release from the supernatant of lymph node cells from infected BALB/c mice treated with UTP. Data correspond to the mean ± SEM values of *n* = 6–5 mice per group. *P < 0.05 vs. untreated (by Student’s *t*-test).

To investigate the “cross-talk” between P2X7 and P2Y receptors in the course of *L. amazonensis* infection *in vivo*, P2X7^−/−^ and WT mice were infected subcutaneously (in the footpad) with 10^6^ promastigotes of *L. amazonensis*, and then treated with six shots of UTP at the infected footpad, at intervals of 3 at 4 days, from 7 days post-infection. Lesion size (swelling) was monitored throughout the infection course, and mice were euthanized 26 days post-infection (Figure 7A), for parasite load, cytokine, and ATP release analysis of infected tissues. We observed that leishmanial lesions were considerably smaller in WT mice that received UTP, when compared with vehicle-treated (PBS) mice (Figure 7B). However, this effect was absent in P2X7^−/−^ mice, where UTP treatment failed to reduce lesion size relative to the vehicle-treated control (Figure 7F).

To examine in more detail the effect of UTP *in vivo*, we estimated the number of parasites present in inflammatory sites (footpad and popliteal lymph node) by the LDA. Strikingly, in mice lacking the P2X7 receptor, the parasite load did not decrease significantly as a response to UTP treatment, which decreased significantly the parasite load both in the footpad and in the lymph node, in WT mice (Figures 7C,D). In addition, we detected an increase in cellularity in the footpad after UTP treatment, in WT mice only (Figure 7E).

IL-1β production *via* the NLRP3 inflamassome activates macrophages to produce microbicidal compounds such as ROS and NO (34), in a mechanism mediated by extracellular ATP (26). In addition, IL-1β promotes Th17 cell differentiation (35), and interleukin-IL-17A (IL-17A) produced by Th17 cells has key roles in the immune response against *Leishmania* (36). Therefore, we evaluated if UTP treatment modulated the production of IL-1β and IL-17A in our experimental model, in a manner dependent on the P2X7 receptor. Lymph node cells from infected WT mice treated with UTP produced higher levels of both IL-1β and IL-17A, compared with unstimulated cells, and this effect was absent in UTP-treated P2X7^−/−^ mice (Figures 7J–K). We also examined ATP release by lymph node cells, in UTP-treated mice during infection with *L. amazonensis*, to confirm the existence of a “cross-talking” between the effects of UTP and ATP− *via* P2Y activation, *in vivo*. We observed that lymph node cells from UTP-treated WT mice secreted significantly higher levels of ATP than those from PBS-treated mice (Figure 7L), indicating that UTP treatment leads to increased ATP production *in vivo*, which contributes to the induction of pro-inflammatory cytokines important for infection control.

## Discussion

**Figure 8:**
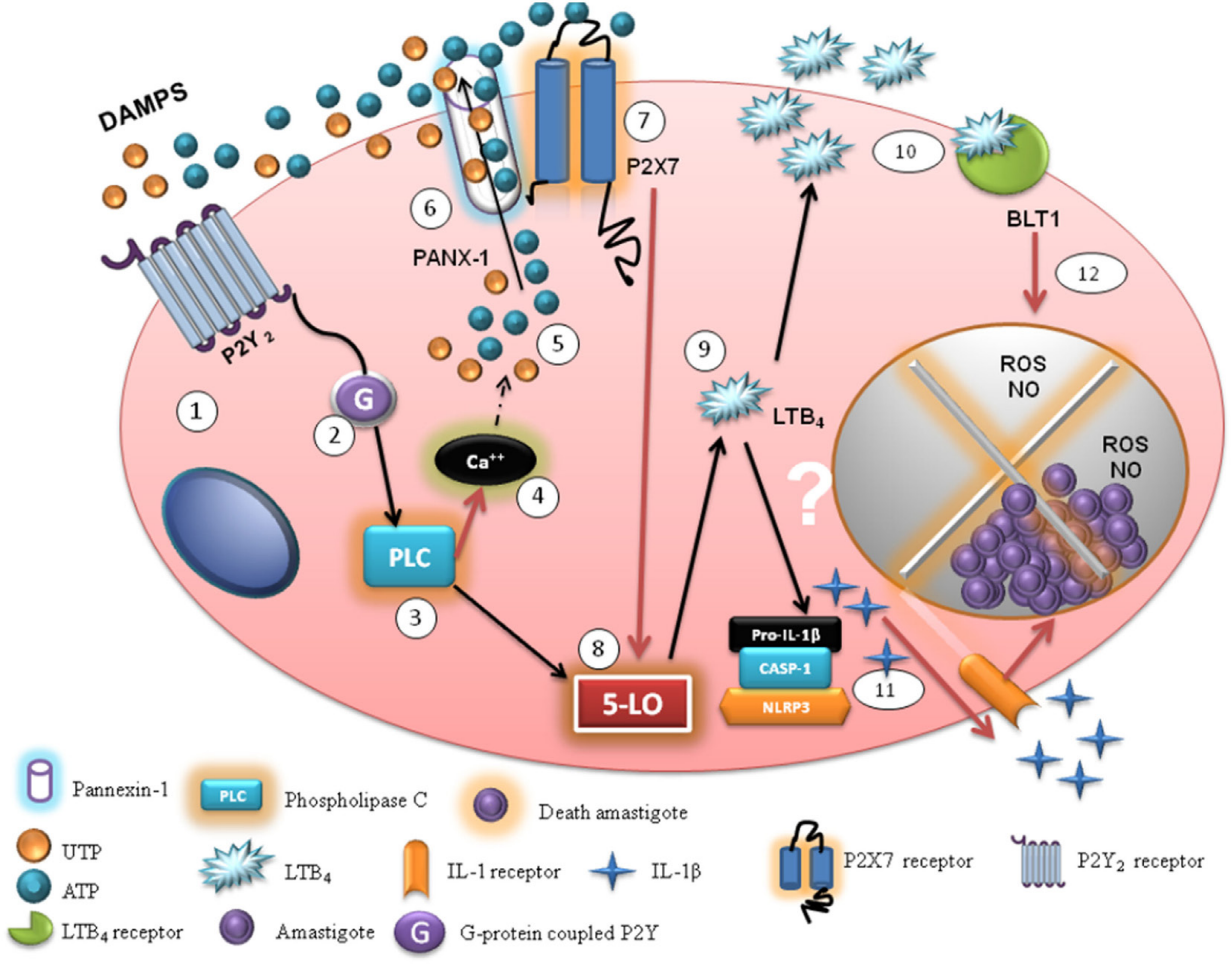
Model of the “cross-talk” between purinergic receptors during the uridine triphosphate (UTP)-mediated control of *Leishmania amazonensis* infection. We propose a model whereby UTP induces the control of *L. amazonensis* infection by a “cross-talk” between P2Y_2_ and P2X7 receptors, activated by eUTP and eATP. During *L. amazonensis* infection, P2Y_2,4_ and P2X7 are upregulated in macrophages (1), which allows eATP and eUTP to activate an important mechanism of infection control *via* P2 receptors. Initially, lower levels of eATP and eUTP [damage-associated molecular patterns (DAMPs)] activate the P2Y_2_ receptor (1) and stimulate its G_q_ coupled protein (G) (2), activating the phospholipase C—IP_3_ (PLC, phosphatidil-inositol 1–3 phosphate) pathway (3) that modulates calcium (Ca^++^) release from the endoplasmic reticulum (4). We suggest that eATP and eUTP potentiate ATP release *via* P2Y_2_ activation, triggering further phospholipase C and Ca^++^ mobilization, which recruits PANX-1 channels that releasing more DAMPs such as adenosine triphosphate (5), acting mainly *via* P2Y_2_ and P2X7 signaling (6, 7). The combined innate response of both P2 receptors triggers reactive oxygen species (ROS) and NO production (12), and activation of the enzyme 5-LO further culminates in LTB4 production (9, 10, 11) and IL-1β secretion, in an NLRP3 inflammasome-dependent manner (11). During chronic infection with *L. amazonensis*, the mediators mentioned above help to induce helper T lymphocytes such as Th1 and Th17 cells, which produce IFN-γ and IL-17 to further activate anti-leishmanial mechanism in phagocytes (such as NO and ROS production). Thus, P2Y and P2X activation cooperate in boosting immune responses against *L. amazonensis*. Black arrows indicate pathways supported by data in this paper; dotted black arrows represent potential pathways (based on data from other systems) and red arrows represent pathways supported by data from previous studies.

Our group has been reporting the involvement of extracellular nucleotides such as UTP and ATP in restraining the replication of *L. amazonensis* (5, 19, 20, 27). Recent evidence suggests that the presence of ATP in the extracellular environment can trigger further ATP release for different human cell types—including leukocytes, urothelial cells, osteocytes, neutrophils, and macrophages (37, 38). Also, UTP induces ATP release during different inflammatory conditions, mediated by P2 receptors (8, 31, 39). In the present study, we show that different P2 receptors—activated by eUTP and eATP—have key roles in experimental cutaneous leishmaniasis. Our data also reveal an important “cross-talk”—based on eATP release amplification—between P2Y_2_ and P2X7 receptors in activation of anti-leishmanial strategies in mice (Figure 8).

Importantly, we show that the reduction in *L. amazonensis* infection mediated by P2Y_2_ receptors can be achieved by treatment with low eATP concentrations (50–100 µM) implying the involvement of P2Y_2_ receptor; fact reinforced by the use of a P2Y_2_ antagonist (Figures 1 and 2). Furthermore, we found that UTP treatment increased ATP release by macrophages infected with *L. amazonensis*, but this effect was not observed in untreated cultures (Figure 4). Thus, our results indicate that the activation of P2Y_2_ receptors by eATP/eUTP leads to further ATP release, by PANX-1-dependent mechanism, which in turns activates P2X7 receptors—amplifying the microbial capacity of macrophages (Figures 3 and 4). Interestingly, the lack of eATP-mediated infection control after pharmacological P2Y_2_ inhibition shows that the effects of eUTP and eATP are both initiate and dependent on P2Y_2_-signaling, suggesting that receptor activation by distinct DAMPs (UTP and ATP) is required to reduce *L. amazonensis* load in macrophages (Figure 2).

Marques-da-Silva and colleagues (19) proposed that a possible heteromeric association between P2Y_2_ and P2Y_4_ during *L. amazonensis* infection may be responsible for antiparasitic responses mediated by eUTP in infected cells. Here, we shown that P2X7 receptor is required to effects antiparasitic induced by P2Y_2_ activation; particularly in *L. amazonensis* infected macrophages, the P2X7 receptor increased sensitivity to eATP (Figures 3B–D) and indirectly to eUTP (Figures 3A–C) because we found higher ATP levels in macrophages treated with UTP. Furthermore, we do not exclude that the release of ATP mediated by P2Y_2_ also potentiates the release of UTP helping on maintenance P2Y activation in our system.

The P2X7 receptor has a key role in immune responses against intracellular parasites, modulating macrophage and T cell responses (25, 40). Our data indicate that the P2X7 receptor is crucial for the reduction in *L. amazonensis* infection achieved by UTP treatment, since UTP-mediated parasite control was prevented by pharmacological inhibition or gene knockout of the P2X7 receptor, both *in vitro* and *in vivo* (Figures 3–7). In agreement, we report in 2009 that the P2X7 receptor plays a key role in the control of infection by *L. amazonensis in vitro*; and recently Figliuolo and colleagues (16) reported that P2X7^−/−^ mice are more susceptible to *L. amazonensis* infection. In addition, our group recently showed that P2X7-mediated *L. amazonensis* elimination involves 5-lipoxygenase (5-LO) activation and LTB_4_ secretion in infected macrophages (5). Furthermore, LTB_4_ upregulates effectors mechanisms (such as the phagocytic capacity) in macrophages and neutrophils and stimulates ROS generation in *L. amazonensis* infection (41). In present study, we show that the anti-leishmanial effects of UTP are abolished in 5-LO deficient mice, and that LTB_4_ production is mediated by eUTP in macrophages infected with *L. amazonensis*, in a P2X7-dependent manner. These combined data suggest that P2Y_2_ activation by eUTP/eATP causes ATP release, which then triggers P2X7-dependent LTB_4_ production, *via* 5-LO activation.

In the immune system, ATP triggers mainly pro-inflammatory reactions, such as the release of IL-1β, phagocytosis, chemotaxis, and cell adhesion to endothelia (42). The release of ATP activates the P2X7 receptor, which triggers an immediate (within milliseconds) opening of the ATP-gated P2X7 channels permeable to small cationic ions (43). Subsequently, PANX-1 hemichannels are recruited and activated, allowing the passage of larger anionic molecules of up to 900 Da, such as ATP (44, 45). Our experiments indicate that, as well as the P2Y_2_ and P2X7 receptors, PANX-1 channels also have a key function in evoking an antiparasitic immune response after UTP treatment, because UTP-mediated anti-leishmanial activities were abolished by PANX-1 pharmacological inhibition (with CBX) or gene knockout (PANX-1^−/−^ macrophages). Other studies have proposed the involvement of PANX-1 channels in mediating ATP secretion in professional phagocytes (38), and THP-1 monocytes require PANX-1 to secreted nucleotides, which represent *eat-me* and *find-me* signals (8). Although PANX-1 channels appears to be the central mechanism for ATP release after UTP treatment in our system, ATP exocytosis may also contribute to this, because the reduction in infection by UTP treatment was partially disrupted by actin cytoskeleton and vesicle traffic inhibition (using cytochalasin A and brefeldin A, respectively).

We show here that UTP-treatment induces a strong immune response against *L. amazonensis in vivo*, in which the P2X7 receptor is an essential player (Figures 7B–F). ATP released by migrating cells can positively modulate ATP-releasing cells (46). The contribution of migrated cells to eATP release can occur *in vivo* after *L. amazonensis* infection, since an increase in the number of footpad cells was observed (Figure 6). IL-1β and IL-17 cytokines perform important functions in potentiating inflammatory reactions (47, 48), both cytokines are required to triggered NO production in macrophages infected with *Leishmania* (34, 36). In this scenario, we observed IL-1β and IL-17 production in response to UTP, phenomena absent in P2X7^−/−^. This impaired cytokine response in P2X7^−/−^ mice might contribute to the susceptibility observed in this strain since NLRP3 inflamassome-derived IL-1β restricts *L. amazonensis* proliferation in macrophages and contributes to generation of IL-17 producing Th17 cells (35, 40). In addition, the reduced ATP released observed in P2X7^−/−^ mice might impairs the neutrophil recruitment and the neutrophil-mediated IL-17 production in the site of infection.

In addition, the activation of P2Y_2_ and P2X7 receptors induces chemotaxis, leukocyte migration, and cytokine secretion in several models of inflammatory response (38, 40, 42). The P2X7 receptor has been implicated in controlling infection by intracellular microorganism *via* a Th1/Th17 immune response, dependent on the NLRP3 inflammasome (40). Interestingly, the induction of innate cytokines related to the Th17 profile during *L. infantum* infection depends on 5-LO and LTB_4_ (49), and the immune response mediated by P2X7 activation requires the BLT1 (LTB_4_ receptor) receptor in macrophages infected with *L. amazonensis* (5). Moreover, IL-17 is produced in a P2X7-dependent manner during *L. amazonensis* infection (16), suggesting that the production of IL-17 is elicited by 5-LO activation as well as the production of LTB_4_ and IL-1β, in our experimental model. Conversely, P2Y_6_ and P2X7 receptors have been implicated in lymphocyte activation (50), and the P2Y_12_ receptor-triggered IL-17 production and Th17 differentiation in a model of experimental autoimmune encephalomyelitis in mice (51). In conclusion, our data reinforces that P2Y and P2X receptors have key roles in mediating immune responses in several inflammatory models (52).

When combined with previous observations, our data suggest that the IL-1β and IL-17 production observed in UTP-treated mice might be a consequence of ATP release and LTB_4_ production mediated by P2X7 activation during *L. amazonensis* infection. At the site of infection, IL-1β and IL-17 triggers the recruitment of immune cells, and microbial responses (such as ROS and NO production) that are detrimental to the *Leishmania*. The results shown here could be used for designing new strategies against Leishmaniasis using therapeutic approaches activating P2Y and P2X7 receptors.

## Ethics Statement

The procedures for the care and use of animals were according to the guidelines of the Brazilian College of Animal Experimentation (COBEA). All efforts were made to minimize animal suffering and to reduce the number of animals used in this study. This study was approved and followed all the guidelines established by the Ethics Committee on the Use of Animals (CEUA) of the Biophysic Institute Carlos Chagas Filho (IBCCF, UFRJ, no. 082/15).

## Author Contributions

MT, MF, and RC-S designed and performed the experiments; NA performed LTB_4_ measure. FM, CC, and JF contributed reagents/materials, analyzed results, and revised the manuscript. RC-S and MT analyzed the data and wrote the manuscript.

## Conflict of Interest Statement

The authors declare that the research was conducted in the absence of any commercial or financial relationships that could be construed as a potential conflict of interest. The reviewer DN declared a shared affiliation, though no other collaboration, with several of the authors MT, MF, NA, CC, and RC-S to the handling Editor.
